# Clinical subtypes of chronic traumatic encephalopathy: literature review and proposed research diagnostic criteria for traumatic encephalopathy syndrome

**DOI:** 10.1186/s13195-014-0068-z

**Published:** 2014-09-24

**Authors:** Philip H Montenigro, Christine M Baugh, Daniel H Daneshvar, Jesse Mez, Andrew E Budson, Rhoda Au, Douglas I Katz, Robert C Cantu, Robert A Stern

**Affiliations:** 1 Department of Anatomy and Neurobiology, Boston University School of Medicine, 72 East Concord Street, Boston 02118, MA, USA; 2 Department of Neurology, Boston University School of Medicine, 72 East Concord Street, Boston 02118, MA, USA; 3 Behavioral Neurosciences Program, Boston University School of Medicine, 72 East Concord Street, Boston 02118, MA, USA; 4 BU Alzheimer’s Disease Center, Boston University School of Medicine, 72 East Concord Street, Boston 02118, MA, USA; 5 VA Boston Healthcare System, 150 S Huntington Avenue, Boston 02130, MA, USA; 6 Framingham Heart Study, National Heart, Lung and Blood Institute, 72 East Concord Street, Boston 02118, MA, USA; 7 Braintree Rehabilitation Hospital, 250 Pond Street, Braintree 02184, MA, USA; 8 Department of Neurosurgery, Boston University School of Medicine, 73 East Concord Street, Boston 02118, MA, USA; 9 Department of Neurosurgery, Emerson Hospital, 131 Old Road, Suite 820, Concord 01742, MA, USA

## Abstract

The long-term consequences of repetitive head impacts have been described since the early 20th century. Terms such as punch drunk and dementia pugilistica were first used to describe the clinical syndromes experienced by boxers. A more generic designation, chronic traumatic encephalopathy (CTE), has been employed since the mid-1900s and has been used in recent years to describe a neurodegenerative disease found not just in boxers but in American football players, other contact sport athletes, military veterans, and others with histories of repetitive brain trauma, including concussions and subconcussive trauma. This article reviews the literature of the clinical manifestations of CTE from 202 published cases. The clinical features include impairments in mood (for example, depression and hopelessness), behavior (for example, explosivity and violence), cognition (for example, impaired memory, executive functioning, attention, and dementia), and, less commonly, motor functioning (for example, parkinsonism, ataxia, and dysarthria). We present proposed research criteria for traumatic encephalopathy syndrome (TES) which consist of four variants or subtypes (TES behavioral/mood variant, TES cognitive variant, TES mixed variant, and TES dementia) as well as classifications of ‘probable CTE’ and ‘possible CTE’. These proposed criteria are expected to be modified and updated as new research findings become available. They are not meant to be used for a clinical diagnosis. Rather, they should be viewed as research criteria that can be employed in studies of the underlying causes, risk factors, differential diagnosis, prevention, and treatment of CTE and related disorders.

## Introduction

Chronic traumatic encephalopathy (CTE) is a neurodegenerative disease characterized by the accumulation of hyperphosphorylated tau protein (p-tau) in neurons and astrocytes in a pattern that is unique from that of other tauopathies, including Alzheimer’s disease (AD) and frontotemporal lobar degeneration. The p-tau deposition initially occurs focally, as perivascular neurofibrillary tangles and neurites at the depths of the cerebral sulci. It spreads to involve superficial layers of adjacent cortex, eventually resulting in widespread degeneration of the medial temporal lobes, frontal lobes, diencephalon, and brainstem [1],[2]. Unlike AD, there is a paucity of beta amyloid neuritic plaques. CTE has been found most often in professional athletes involved in contact sports (for example, boxing and American football) who have been subjected to repetitive head blows resulting in concussive and subconcussive trauma [3],[4]. Neuropathologically confirmed CTE has been reported in individuals as young as 17 and in athletes who played sports only through high school or college. It also has been found in non-athletes who have experienced repetitive head impacts, including epileptics, developmentally disabled individuals who head-bang, and victims of physical abuse [2]. Moreover, CTE has been neuropathologically diagnosed in military service members previously deployed in Iraq and Afghanistan with histories of repetitive brain trauma [2],[5]. At this time, it is not completely clear whether all cases of neuropathologically confirmed CTE would demonstrate a progressive course if they lived long enough.

All cases of neuropathologically confirmed CTE reported to date have had a history of repetitive head impacts, although there has been some suggestion that a single traumatic brain injury (TBI) may also lead to the neuropathological changes of CTE [6]. Although head impacts appear to be necessary for the initiation of the pathogenetic cascade that eventually leads to neurodegeneration, the history of head impacts is not sufficient and additional risk factors (including genetic susceptibility markers) remain unknown. The incidence and prevalence of CTE are also unknown, although the number potentially affected could be quite large. Every year, between 1.6 and 3.8 million individuals in the US experience a sports-related concussion [7], and the number of youth sports-related concussions has grown in recent years [8]. The incidence of repetitive subconcussive blows (that is, hits to the head that produce enough force to hamper neuronal integrity but that do not result in clinical concussion symptoms) is much greater [9]. For example, a study by Broglio and colleagues [10] found that, per season, high school football players receive an average of 652 head blows that exceed 15 g of force. With over 1 million high school students playing American football each year and with the size and speed of football players increasing [11], the public health impact of CTE may be quite significant in future years.

*In vivo* diagnosis of CTE is needed to conduct research on risk factors and epidemiology and to perform clinical trials for prevention and treatment. Sensitive and specific biomarkers for CTE are being developed and include structural and neurochemical imaging techniques and positron emission tomography (PET) with new ligands that selectively bind to p-tau [4],[12],[13]. These approaches hold promise to detect underlying neuropathological changes of CTE. However, the clinical features directly associated with these changes have only recently been described and have been based on retrospective reports of family members of deceased individuals who received a neuropathological diagnosis of CTE [2],[14].

In a recent article from our group [14], we examined the clinical presentation of 36 adult males selected from all cases of neuropathologically confirmed CTE at the Boston University Center for the Study of Traumatic Encephalopathy Brain Bank. The cases were all athletes, had no comorbid neurodegenerative or motor neuron disease, and had family member informants who provided retrospective reports of history and clinical features. The semi-structured ‘psychological autopsies’ were conducted blind to the subjects’ neuropathological findings. Three of the 36 subjects were asymptomatic. In the remaining 33 symptomatic subjects, a triad of cognitive, behavioral, and mood impairments was found, and cognitive changes were reported for almost all subjects at some time in the course of disease. However, two relatively distinct clinical presentations emerged: one group had initial features involving behavior (that is, explosivity, physical and verbal violence, being ‘out of control’, and impulsivity) or mood (that is, depression and hopelessness) or both (n = 22), and another group had initial features involving cognition (that is, episodic memory impairment, executive dysfunction, poor attention, and concentration) (n = 11). Symptom onset for the ‘behavior/mood group’ occurred at a significantly younger age than for the ‘cognition group’. Most subjects in the behavior/mood group eventually developed cognitive difficulties, although significantly fewer subjects in the cognition group eventually demonstrated behavioral and mood changes. Significantly more subjects in the cognition group developed dementia than those in the behavior/mood group. Less than one third of the sample had reported motor features, including parkinsonism.

Although the study by Stern and colleagues [14] involved the largest case series to date of neuropathologically confirmed cases of CTE without comorbid conditions and with clinical histories, the sample size was small and the generalizability of the findings was hampered by the potential bias of a sample composed of former athletes whose family members agreed to their brain donation. This limitation notwithstanding, the finding of two possible clinical subtypes of CTE was consistent with previous literature. In the present article, we provide a review of the world’s literature on the clinical features exhibited by athletes with histories of repetitive head impacts. After the literature review, we provide proposed research diagnostic criteria for ‘traumatic encephalopathy syndrome’ (TES), derived from this literature review and from our own research into the clinical presentation of CTE [1],[2],[14]. As described below, these criteria are meant to initially characterize what is known to date and provide a foundation for developing more precise clinical criteria informed by ongoing and future research and clinical review.

## Historical terms for chronic traumatic encephalopathy

In his seminal 1928 article in the *Journal of the American Medical Association*, Martland [15] used the term ‘punch drunk’ to describe boxers suffering from symptoms he believed to be related to the repetitive blows they received in the ring. Since that time, various terms have been used to describe the clinical syndrome associated with repetitive head impacts, predominantly in studies of boxers. In 1934, Parker [16] published an article in which he referred to the ‘traumatic encephalopathy of pugilists’. In 1937, Millspaugh [17] first used the term ‘dementia pugilistica’, which is still used by clinicians and researchers. Other terms coined through the decades include ‘traumatic encephalitis’ [18], ‘cumulative encephalopathy of the boxer’ [19], ‘psychopathic deterioration of pugilists’ [20], ‘chronic boxer’s encephalopathy’ [21], and ‘traumatic boxer’s encephalopathy’ [22]. In 1949, Critchley first used the designation ‘chronic traumatic encephalopathy’ [23], or CTE, but later modified it to ‘chronic progressive traumatic encephalopathy’ [24] because several cases apparently progressed from an early mild state to severe dementia [23]-[25]. Johnson [26] suggested that the latter term erroneously implies that progression is inevitable. In his case series, little to no deterioration is reported in half of the cases followed for 5 years. In recent reviews of the literature, Victoroff (alone [27] and with Baron [28]) suggested using the more general and inclusive term ‘traumatic encephalopathy’.

In 2005, Omalu and colleagues [29] described the first case of neuropathologically confirmed CTE in an American football player. Since that time, there has been increasing public attention to this disease, and reports of CTE in deceased football players, including several well-known athletes, have prompted a tremendous focus on what is commonly referred to as football’s ‘concussion crisis’. The scientific community also has become dramatically more aware of CTE since it was discovered in American football players. For example, a PubMed search using the terms ‘chronic traumatic encephalopathy’, ‘traumatic encephalopathy’, ‘dementia pugilistica’, or ‘punch drunk’ resulted in 14 publications in the 5-year period ending in December 2001 compared with 116 publications in the 5-year period ending in December 2013.

## Early concepts regarding subtypes

In a 1950 editorial in the *British Medical Journal*, Jokl [30] stressed that CTE was not a single syndrome but rather two kinds of chronic impairment, with either predominant ‘behavioral-psychopathic or neurological-psychiatric’ features. He described the behavioral-psychopathic subtype as involving ‘viciousness’, ‘murder committed from jealousy’, and delinquency. In contrast, he described the neurological-psychiatric subtype as involving cognitive deficits, dementia, and motor impairment [30]-[32]. Grahmann and Ule [33] (1957) described three general subtypes: (1) a progressive dementia that typically involved cognitive impairment and developed following a latency from the time of boxing retirement, (2) a stable neurological presentation temporally and etiologically related to the head impacts and not representative of a progressive disease, and (3) a paranoid and psychotic subtype absent of cognitive changes. Critchley [23] maintained that there were three commonly recurring presentations of CTE that resembled, but could be distinguished from, (1) neurosyphilis (for example, psychopathy, altered personality, and later dementia), (2) multiple sclerosis (for example, scanning speech, tremor, and progressive cognitive decline), and (3) frontal lobe tumor (for example, executive impairments, headache, and eye ache). He later added a fourth presentation: striatal parkinsonian (for example, masked facial features and tremor) [24]. In a study of 17 retired boxers, Johnson [26] described four different ‘organic psychosyndromes’: cognitive problems with progressive dementia, behavioral issues related to ‘morbid jealousy’, behavioral issues related to rage and personality disorders, and mood and behavioral disturbance related to persistent psychosis.

## Literature search methods

To examine previous literature describing the clinical presentation of CTE associated with exposure to head impacts through sports participation, we conducted a literature search using PubMed, PubMed Central, and Medline databases. Search terms included ‘chronic traumatic encephalopathy’, ‘punch drunk’, ‘traumatic encephalopathy’, ‘dementia pugilistica’, ‘chronic boxer’s encephalopathy’, ‘chronic progressive traumatic encephalopathy’, ‘psychopathic deterioration of pugilists’, and ‘repetitive brain injury’. In addition, bibliographies of recent literature reviews were cross-referenced [1],[27],[34]-[39]. It should be noted that most online databases are limited to articles published since the 1950s. Because essential work in this field began in 1928, archival research was carried out by hand, and international works were obtained with assistance from the Boston University Medical Library Interlibrary Loan Department. Materials retained included articles, reviews, dissertations, society transactions, association reports, and book chapters. To be reasonably confident about the diagnoses used, several criteria were used to determine inclusion in this review: (1) only case series, and not individual case reports, were included; (2) adequate information must be provided in the report to allow classification of cases as confirmed CTE, probable CTE, or possible CTE by using Jordan’s criteria [35],[40],[41]; and (3) only cases involving athletes were included.

## Results of literature review

Following the exclusion of articles and cases that did not meet the above criteria, the literature review resulted in 202 cases from 20 published case series, four books, and one medical dissertation. The cases are summarized in Table 1[2],[16],[22]-[26],[29],[31]-[33],[42]-[54]. Nineteen cases were published before 1950, 29 cases were published in the 1950s, 49 were published in the 1960s, 13 were published in the 1970s, four were published in the 1980s, 19 were published in the 1990s, and 69 were published since 2000. Using Jordan’s criteria [35], we approximated that 29 would have possible CTE, 90 would have probable CTE, and 83 would have definite CTE. Of the entire sample, 141 were boxers, 54 were American football players, five were ice hockey players, and two were professional wrestlers. The clinical features described in all of the cases were classified into one of four categories: behavioral, mood, cognitive, and motor. Table 2 summarizes the clinical features most commonly described across all cases. In 68% of cases, the course of the clinical syndrome was described as progressive. In cases in which a distinction in clinical syndrome was made, the behavioral and mood features were reported to be more stable, whereas the cognitive features were described as progressive, often resulting in dementia. Compared with cases described as progressive, cases described as stable were substantially younger. A large number of cases had a period of latency of several years between the end of exposure to head impacts and the presentation of clinical signs and symptoms. In neuropathologically confirmed cases, authors described the initial clinical presentation as involving mood or behavioral disturbance (or both) without cognitive impairment in 28%, as having cognitive impairment without concurrent mood or behavioral difficulties in 32%, and as having initial mixed cognitive and mood/behavioral disturbance in 40%.

**Table 1:** Summary of published cases describing the clinical features of chronic traumatic encephalopathy

		**Clinical features**			
**Study**	**Sample**	**Behavioral**	**Mood**	**Cognition**	**Motor**
	**(total n = 202)**				
Parker [16] (1934)	Boxers (n = 3)	Social	Anxiety	Reduced intelligence	Ataxia
		inappropriateness	Labile emotions	Memory impairment	Clonus
		Childish behavior	Fatigue	Impaired attention	Dragging gait
				Visuospatial difficulties	Dysarthria
					Muscle weakness
					Spasticity
					Tremor
Herzog [42] (1938)	Boxers (n = 7)	Boastfulness	Apathy	General cognitive impairment	Dysarthria
		Personality changes	Flat affect	Memory difficulties	Masked facies
		Impulsiveness		Perseveration	Shuffling gait
		Loss of control		Language difficulties	Truncal ataxia
				Alogia	
				Dementia	
Knoll *et al*. [43] (1938)	Boxers (n = 3)	Personality changes	Apathy	General cognitive impairment	Ataxia
			Flat affect	Memory impairment	Dysarthria
			Loss of interest	Visuospatial difficulties	Masked facies
			Prolix	Alogia	
				Dementia	
Jokl [31] (1941) and Jokl and Guttmann [32] (1933)	Boxers (n = 3)	Boastfulness	Apathy	Reduced intelligence	Ataxia
		Childish behavior	Depression	Executive dysfunction	Dysarthria
		Paranoid delusions	Euphoria	Memory impairment	Masked facies
		Personality changes	Fatigue	Impaired attention	Muscle weakness
		Physical violence	Flat affect	Altered concentration	Shuffling gait
		Psychosis	Insomnia	Language difficulties	Tremor
		Short fuse	Irritability	Dysgraphia	Unsteady gait
		Explosivity	Labile emotions	Visuospatial difficulties	
		Social inappropriateness	Loss of interest		
		Verbal violence	Mania		
			Mood swings		
Schwarz [44] (1953)	Boxers (n = 3)	Personality changes	Fearfulness	Memory impairment	Ataxia
		Short fuse	Irritability	Altered concentration	Dysarthria
		Explosivity	Labile emotions	Language difficulties	Masked facies
					Muscle weakness
					Stamping gait
					Tremor
					Unsteady Gait
Soeder and Arndt [45] (1954)	Boxers (n = 5)	Boastfulness	Apathy	General cognitive impairment	Clonus
		Disinhibited behavior	Depressed mood	Executive dysfunction	Dysarthria
		Inappropriate speech	Euphoria	Memory impairment	Masked facies
		Paranoia	Fatigue	Impaired attention	Rolling gait
		Personality changes	Flat affect	Altered concentration	Tremor
		Physical violence	Insomnia	Language difficulties	Truncal ataxia
		Psychosis	Mania	Alogia	Unsteady gait
		Short fuse	Mood swings		
		Explosivity	Prolix		
		Social inappropriateness			
Grahmann and Ule [33] (1957)	Boxers (n = 4)	Childish behavior	Apathy	General cognitive impairment	Dysarthria
	Confirmed CTE (1)	Disinhibited behavior	Depressed	Executive dysfunction	Swaying gait
		Disinhibited speech	Euphoria	Memory impairment	Masked facies
		Impulsivity	Labile emotions	Impaired attention	
		Loss of control	Fatigue	Altered concentration	
		Physical violence	Flat affect	Dementia	
		Personality changes	Irritable		
		Short fuse	Mood swings		
		Explosivity	Prolix		
		Social inappropriateness			
Muller [46] (1958)	Boxers (n = 3)	Social isolation	Fatigue	General cognitive impairment	Dysarthria
		Personality changes	Irritability	Executive dysfunction	Unsteady gait
		Lack of insight		Impaired attention	Spastic gait
				Memory impairment	
				Altered concentration	
				Dementia	
Spillane [47] (1962)	Boxers (n = 5)	Childish behavior	Anxiety	General cognitive impairment	Ataxia
		Disinhibited behavior	Depressed mood	Reduced intelligence	Dysarthria
		Impulsivity	Euphoria	Memory impairment	Dragging gait
			Mania	Visuospatial difficulties	Muscle weakness
			Payne mood swings	Dysgraphia	Tremor
				Lack of insight	Unsteady gait
				Dementia	
Mawdsley and Ferguson [22] (1963)	Boxers (n = 10)	Impulsivity	Apathy	General cognitive impairment	Ataxia
		Loss of control	Depression	Reduced intelligence	Dysarthria
		Physical violence	Insomnia	Memory impairment	Dragging gait
		Psychosis	Irritability	Language difficulties	Masked facies
		Paranoid delusions		Dysgraphia	Muscle weakness
		Personality changes		Dementia	Tremor
		Short fuse			Unsteady gait
		Explosivity			
		Social inappropriateness			
		Verbal violence			
Critchley [23]-[25] (1949, 1957, 1964)	Boxers (n = 17)	Disinhibited speech	Depressed	General cognitive impairment	Ataxia
		Disinhibited behavior	Labile emotions	Reduced intelligence	Clumsy
		Impulsivity	Euphoria	Memory impairment	Dysarthria
		Lack of insight	Insomnia	Impaired attention	Masked facies
		Physical violence	Irritable	Altered concentration	Muscle weakness
		Personality changes	Loss of interest	Visuospatial difficulties	Tremor
		Social inappropriateness	Fatigue	Dementia	Unsteady gait
		Short fuse			
Payne [48] (1968)	Boxers (n = 6)	Disinhibited behavior	Depressed mood	General cognitive impairment	Ataxia
		Impulsivity	Labile emotions	Reduced intelligence	Dysarthria
		Paranoid delusions	Insomnia	Altered concentration	Unsteady gait
		Physical violence	Mania	Visuospatial difficulties	
		Psychotic	Mood swings	Memory impairment	
		Verbal violence	Suicidal ideation		
Johnson [26] (1969)	Boxers (n = 17)	Loss of control	Anxiety	General cognitive impairment	Ataxia
		Paranoid delusions	Labile emotions	Reduced intelligence	Dysarthria
		Personality changes	Irritability	Memory impairment	Tremor
		Psychotic		Dementia	Dragging gait
		Short fuse			Masked facies
		Explosivity			Muscle weakness
		Verbal violence			
Roberts [49] (1969)	Boxers (n = 11)	Lack of insight	Apathy	Reduced intelligence	Ataxia
		Paranoid delusions	Depression	Executive dysfunction	Dysarthria
		Psychosis	Euphoria	Memory impairment	Dragging gait
		Short fuse	Flat affect	Perseveration	Masked facies
		Explosivity	Labile emotions	Impaired attention	Muscle weakness
				Altered concentration	Shuffling gait
				Language difficulties	Spasticity
				Dysgraphia	Tremor
				Visuospatial difficulties	Unsteady gait
				Dementia	
Corsellis *et al*. [50] (1973)	Boxers (n = 13) Confirmed CTE (13)	Childish behavior	Anxiety	General cognitive impairment	Ataxia
		Paranoid delusions	Labile emotions	Reduced intelligence	Dysarthria
		Personality changes	Irritability	Memory impairment	Masked facies
		Short fuse		Dementia	Muscle weakness
		Explosivity			Tremor
		Social inappropriateness			Staggering gait
		Social isolation			Shuffling gait
		Verbal violence			Unsteady gait
Sabharwal *et al*. [51] (1987)	Boxers (n = 4)	Inappropriate speech	Depression	Reduced intelligence	Ataxia
			Irritability	Memory impairment	Spasticity
			Labile emotions		Dysarthria
			Mood swings		
Jordan *et al*. [52] (1997)	Boxers (n = 19)	Disinhibited speech	Depression	Impaired attention	Ataxia
		Disinhibited behavior	Irritability	Altered concentration	Clonus
			Flat affect	Memory impairment	Dysarthria
			Mania		Spasticity
					Tremor
					Unsteady gait
Omalu *et al*. [29],[53],[54] (2005, 2006, 2010)	Football and wrestling (n = 5)	Paranoid delusions	Suicidality	General cognitive impairment	-
		Social isolation	Anxiety	Memory impairment	
		Physical violence	Labile emotions	Language difficulties	
	Confirmed CTE (5)		Irritability	Executive dysfunction	
			Insomnia	Impaired attention	
			Depression		
Mckee *et al*. [2] (2013)	Boxing, American football, ice hockey, wrestling (n = 64)	Explosivity	Depression	Memory impairment	Dysarthria
		Aggression	Hopelessness	Executive dysfunction	Gait disturbance
		Impulsivity	Suicidality	Impaired attention	Parkinsonism
		Paranoia	Mood swings	Language difficulties	
				Visuospatial difficulties	
				Dementia	
	Confirmed CTE (64)				

CTE, chronic traumatic encephalopathy.

**Table 2:** Summary of clinical features of chronic traumatic encephalopathy found in the literature

**Behavioral features**	**Mood features**	**Cognitive features**	**Motor features**
Explosivity	Depression	Dementia	Ataxia
Loss of control	Hopelessness	Memory impairment	Dysarthria
Short fuse	Suicidality	Executive dysfunction	Parkinsonism
Impulsivity	Anxiety	Lack of insight	Gait Disturbance
Aggression	Fearfulness	Perseveration	Tremor
Rage	Irritability	Impaired attention and	Masked facies
Physical violence	Labile emotions	concentration	Rigidity
Verbal violence	Apathy	Language difficulties	Muscle weakness
Inappropriate speech	Loss of interest	Dysgraphia	Spasticity
Boastfulness	Fatigue	Alogia	Clonus
Childish behavior	Flat affect	Visuospatial	
Social inappropriateness	Insomnia	difficulties	
Disinhibited speech	Mania	General cognitive impairment	
Disinhibited behavior	Euphoria	Reduced intelligence	
Paranoid delusions	Mood swings		
Personality changes	Prolix		
Psychosis			
Social isolation			

In recent years, some authors have made the distinction between ‘classic CTE’ and ‘modern CTE’ [34],[36]. For example, McCrory and colleagues [36] define the classic CTE syndrome based on the clinical descriptions from Roberts [49] and the neuropathological reports from Corsellis and colleagues [50]. Based on these earlier cases of boxers, classic CTE is described as having prominent motor features, including gait disturbance, dysarthria, and pyramidal problems, but without progressive cognitive, behavioral, or mood changes [36]. However, it is important to note that, in his monograph, Roberts [49] clarifies that he is intentionally focusing on the description and quantification of motor signs related to neurological lesions, reducing his focus on ‘the evidence of dementia or personality change’ which he viewed as occurring in a subset of cases [49]. In contrast, ‘modern CTE’ [34],[36], defined as any case report published in 2005 or later, is characterized by predominant mood and behavioral symptoms as well as later progressive cognitive deficits and dementia but with less prevalent motor features. We view this distinction between the earlier and more recent descriptions of the clinical presentation of CTE as largely an artifact of different sources of trauma exposure (that is, predominantly boxers in the ‘classic’ cases and predominantly football players in the ‘modern’ cases).

To explore this issue, we examined further the cases of neuropathologically confirmed pure CTE described in the series of McKee and colleagues [2] and compared the presence of motor features reported for the deceased professional boxers with those reported for the professional football players. The percentage of professional boxers with motor features (71%) far exceeded that of professional football players (13%). Additionally, it was found that in cases with the most advanced stage of CTE neuropathology, there was a striking difference in the presence of cerebellar pathology in professional boxers (83%) and professional football players (57%). The likely cause of this may be related to the differences in the biomechanics of the head trauma that is experienced through the practice of these two different sports [14].

## Previously published diagnostic criteria

To date, there have been two published sets of diagnostic criteria for the clinical diagnosis of CTE. The first diagnostic criteria, proposed by Jordan [35],[40],[41], were developed specifically to represent the likelihood of underlying CTE neuropathology. As such, the following four diagnostic classifications are used: (1) definite CTE (‘any neurological process consistent with the clinical presentation of CTE along with pathological confirmation’), (2) probable CTE (‘any neurological process characterized by two or more of the following conditions: cognitive and/or behavioral impairment; cerebellar dysfunction; pyramidal tract disease or extrapyramidal disease; clinically distinguishable from any known disease process and consistent with the clinical description of CTE’), (3) possible CTE (‘any neurological process that is consistent with the clinical description of CTE but can be potentially explained by other known neurological disorders’), and (4) improbable CTE (‘any neurological process that is inconsistent with the clinical description of CTE and can be explained by a pathophysiological process unrelated to brain trauma’) [35].

In contrast to Jordan’s diagnostic criteria, which are focused on the prediction of underlying CTE neuropathology, the diagnostic criteria of Victoroff [27] are focused on a broad set of clinical signs and symptoms representing a diverse set of possible etiologies and are not meant to predict underlying CTE neuropathology. These provisional research diagnostic criteria for clinically probable traumatic encephalopathy (TE) and clinically possible TE were based on the frequency of clinical symptoms and signs reported in TE case reports published between 1928 and 2010. The Victoroff criteria represent an important addition to the literature but have several limitations. For example, for a diagnosis of clinically probable TE, there is a requirement for two symptoms and three signs. However, there is tremendous overlap and redundancy between the symptoms and the ‘neurobehavioral signs’, including the use of the following terms included as both symptoms and signs: memory loss, irritability, apathy, impulsivity, depression, lability, euphoria, paranoia, and others. Another required criterion for clinically probable TE is the ‘persistence of both symptoms and signs for at least two years after the traumatic exposure’ [27]. This is not consistent with numerous cases of neuropathologically confirmed CTE for which a delayed onset of the clinical presentation is often observed, representing the neurodegenerative nature of the disease [2],[14]. An additional limitation to the Victoroff criteria is the lack of any subtyping of the clinical presentation. That is, the same diagnosis of clinically probable TE could be given to an 80-year-old with memory loss, mental slowing, headache, and nystagmus and to a 22-year-old with depression, anxiety, irritability, and anger. This lack of diagnostic subtyping for a condition with such clinically diverse signs and symptoms would reduce the utility of the criteria for research aimed at elucidating specific clinico-pathological relationships or clinical trials requiring greater specificity of diagnosis to ensure meaningful target outcomes. The criteria are presented in a single table without accompanying descriptive prose, making implementation of the criteria potentially subject to individual interpretation. Finally, the Victoroff criteria do not include or recommend the future use of objective diagnostic tests, such as neuroimaging or other potential biomarkers, to improve upon the diagnostic accuracy, specificity, or ability to detect CTE during life.

## Proposed research diagnostic criteria for traumatic encephalopathy syndrome

We propose research diagnostic criteria that address many of the limitations of the previously published criteria by Jordan [35],[40],[41] and Victoroff [27]. These new criteria are derived from the previous literature on CTE reviewed above as well as the specific findings from the studies by Stern and colleagues [14] and McKee and colleagues [2] on the clinical presentation of neuropathologically confirmed cases of CTE. The term ‘traumatic encephalopathy syndrome’ (TES) was selected instead of ‘chronic traumatic encephalopathy’ (CTE) for the following reasons: (1) we view the designation ‘CTE’ as a neuropathologically defined disease (which is defined by the characteristic deposition of p-tau pathology) rather than a clinical syndrome; (2) TES is meant to describe the clinical presentation of CTE as well as other possible long-term consequences of repetitive head impacts (for example, chronic or progressive axonopathy without tauopathy) but is not meant to include the acute or post-acute manifestations of a single concussion, post-concussion syndrome, or moderate to severe TBI; (3) the use of the word ‘chronic’ in CTE inaccurately connotes a stable condition rather than a progressive disorder [27]; and (4) the inclusion of the term ‘syndrome’ appropriately describes the cluster of clinical features that make up this condition. The proposed research diagnostic criteria for TES include five general criteria, three core clinical features, and nine supportive features that are used to define subtypes of TES: a behavioral/mood variant, a cognitive variant, a mixed variant, and TES dementia. The modifiers ‘progressive course’, ‘stable course’, and ‘unknown/inconsistent course’ are used to describe the clinical course, and if specific motor signs are evident, the modifier ‘with motor features’ is added.

The selection of the five general criteria was based on the literature reviewed above and was designed to favor sensitivity over specificity. This decision is consistent with the previously published diagnostic criteria [27],[35] and is appropriate at this early stage of clinical research into this area. To be included as a core clinical feature, the sign or symptom must have been reported in a minimum of 70% of the cases in the study by Stern and colleagues [14] of neuropathologically confirmed cases of pure CTE. This is in contrast to the algorithm employed in the Victoroff [27] diagnostic criteria for which a sign or symptom was included if it was present in at least 7% of the case reports he reviewed from the literature. The nine supportive features were selected to increase specificity once the general criteria are met and are based on features reported in the previous literature.

The clinical diagnosis of TES is not meant to imply a certainty of underlying CTE neuropathological changes (for example, p-tau accumulation). Rather, TES is meant to be a diagnosis of a clinical syndrome associated with a history of repetitive brain trauma. It is expected that some individuals with TES do indeed have CTE neuropathological changes. However, it is also possible that some individuals with TES have other underlying causes of their clinical presentation, including, but not limited to, progressive white matter degeneration [55] or AD. For this reason, a separate diagnostic classification for ‘possible CTE’, ‘probable CTE’, and ‘unlikely CTE’ is included, based on the presence of additional supportive features, such as biomarkers, which indicate the degree to which the underlying etiology of the clinical presentation of TES is likely due to the CTE pathophysiological process. Finally, we offer six cases (see boxes) as exemplars of the implementation of the TES criteria; each case is a composite of several patients and is created specifically for this purpose.

At this time, risk factors for CTE (above and beyond brain trauma) remain unknown. Among possible variables under investigation by our group and other laboratories are the quantity or severity (or both) of the brain trauma, the initial age and overall duration of head impact exposure, lifestyle factors, and genetic susceptibility. Based on current research findings, it is expected that TES is the clinical manifestation of underlying damage or dysfunction of cortical or subcortical brain structures (or both) and is associated with a history of repetitive brain trauma, including both symptomatic concussions and subconcussive trauma. Although some investigators have suggested that a single moderate to severe TBI may lead to CTE [37] or AD [56] or both, the use of the clinical diagnosis of TES at this time is meant to be used for individuals with repetitive impacts to the head, as defined below. We have included a requirement for a specific minimal amount of exposure to head impacts. This is based on previous findings of post-mortem confirmed CTE cases [1],[2],[5],[50] and will be subject to revisions as additional research is conducted on exposure variables.

## General criteria for traumatic encephalopathy syndrome

All five criteria must be met for a diagnosis of TES:

1. History of multiple impacts to the head (or to the body resulting in impulsive force transmitted to the head). Multiple impacts are defined based upon (a) the types of injuries and (b) the source of exposure.

a. Types of injuries:

i) Mild TBI or concussion, defined according to the Zurich 2012 Consensus Statement on Concussion in Sport [57] as a ‘complex pathophysiological process affecting the brain, induced by biomechanical forces…caused either by a direct blow to the head, face, neck or elsewhere on the body with an “impulsive” force transmitted to the head…the acute clinical symptoms largely reflect a functional disturbance rather than a structural injury and, as such, no abnormality is seen on standard structural neuroimaging studies. Concussion results in a graded set of clinical symptoms that may or may not involve loss of consciousness’. History of this form of trauma can be based on documented records from health-care providers or on self- or informant-reports, after being given an appropriate definition of ‘concussion’ [58]. If there is no reported exposure to other repetitive hits to the head, there should be a minimum of four documented mild TBIs or concussions.

ii) Moderate/severe TBI, defined as having loss of consciousness of at least 30 minutes, alteration of consciousness/mental state of more than 24 hours, post-traumatic amnesia of more than 24 hours, and Glasgow Coma Scale score of less than 13 [59]. If there is no reported exposure to other repetitive hits to the head, there should be a minimum of two moderate/severe TBIs.

iii) ‘Subconcussive’ trauma, defined as biomechanical force to the head or body similar to, or less than, that required for symptomatic concussion but without symptoms and clinical presentation consistent with concussion [3],[4].

b) Source of exposures:

i. Involvement in ‘high exposure’ contact sports (including, but not limited to, boxing, American football, ice hockey, lacrosse, rugby, wrestling, and soccer) for a minimum of 6 years, including at least 2 years at the college level (or equivalent) or higher.

ii. Military service (including, but not limited to, combat exposure to blast and other explosions as well as non-combat exposure to explosives or to combatant or breach training).

iii. History of any other significant exposure to repetitive hits to the head (including, but not limited to, domestic abuse, head banging, and vocational activities such as door breaching by police).

iv. For moderate/severe TBI, any activity resulting in the injury (for example, motor vehicle accident).

2) No other neurological disorder (including chronic residual symptoms from a single TBI or persistent post-concussion syndrome) that likely accounts for all clinical features, although concomitant diagnoses of substance abuse, post-traumatic stress disorder (PTSD), mood/anxiety disorders, or other neurodegenerative diseases (for example, AD and frontotemporal dementia) or a combination of these can be present.

3) Clinical features must be present for a minimum of 12 months. However, if treatment (for example, ‘antidepressant’ medication) results in an improvement in select symptoms, the clinician should use her or his best judgment to decide whether the symptoms would have persisted or progressed if treatment had not been initiated.

4) At least one of the core clinical features must be present and should be considered a change from baseline functioning.

5) At least two supportive features must be present.

## Core clinical features of traumatic encephalopathy syndrome

At least one of the core clinical features must be present:

1) *Cognitive*. Difficulties in cognition:

a) as reported by self or informant, by history of treatment, or by clinician’s report of decline; and

b) substantiated by impairment on standardized mental status or neuropsychological tests of episodic memory, executive function, and/or attention, as defined by scores at a level of at least 1.5 standard deviations below appropriate norms.

2) *Behavioral*. Being described as emotionally explosive (for example, having a ‘short fuse’ or being ‘out of control’), physically violent, and/or verbally violent, as reported by self or informant, by history of treatment, or by clinician’s report. A formal diagnosis of intermittent explosive disorder would meet this criterion but is not necessary.

3) *Mood*. Feeling overly sad, depressed, and/or hopeless, as reported by self or informant, by history of treatment, or by clinician’s report. A formal diagnosis of major depressive disorder or persistent depressive disorder would meet this criterion but is not necessary.

## Supportive features of traumatic encephalopathy syndrome

A minimum of two of the following features must be present for a diagnosis of TES:

1) *Impulsivity*. Impaired impulse control, as demonstrated by new behaviors, such as excessive gambling, increased or unusual sexual activity, substance abuse, excessive shopping or unusual purchases, or similar activities.

2) *Anxiety*. History of anxious mood, agitation, excessive fears, or obsessive or compulsive behavior (or both), as reported by self or informant, history of treatment, or clinician’s report. A formal diagnosis of anxiety disorder would meet this criterion but is not necessary.

3) *Apathy*. Loss of interest in usual activities, loss of motivation and emotions, and/or reduction of voluntary, goal-directed behaviors, as reported by self or informant, history of treatment, or clinician’s report.

4) *Paranoia*. Delusional beliefs of suspicion, persecution, and/or unwarranted jealousy.

5) *Suicidality*. History of suicidal thoughts or attempts, as reported by self or informant, history of treatment, or clinician’s report.

6) *Headache*. Significant and chronic headache with at least one episode per month for a minimum of 6 months.

7) *Motor signs*. Dysarthria, dysgraphia, bradykinesia, tremor, rigidity, gait disturbance, falls, and/or other features of parkinsonism. If present, the modifier ‘with motor features’ should be used (see below).

8) *Documented decline*. Progressive decline in function and/or a progression in symptoms and/or signs, based upon repeated formal testing, clinician examination, or other formal measurement (for example, informant questionnaire) for a minimum of 1 year.

9) *Delayed onset*. Delayed onset of clinical features after significant head impact exposure, usually at least 2 years and in many cases several years after the period of maximal exposure. It should be noted, however, that individual cases may begin to develop the clinical features of TES during their period of head impact exposure (for example, while still actively involved in a collision sport), especially older individuals or those who have been engaged in the high-exposure activity for many years. It may also be difficult to differentiate the clinical presentation of prolonged or persistent post-concussion syndrome (pPCS) from that of TES. Therefore, there could be cases for whom there is overlap of resolving pPCS and the initial features of TES, thus masking any delayed onset of TES.

## Traumatic encephalopathy syndrome diagnostic subtypes}

1) TES behavioral/mood variant (TES-BMv)

a) Behavioral or mood core features (or both) without cognitive core features.

2) TES cognitive variant (TES-COGv)

a) Cognitive core features without behavioral or mood core features (or both).

3) TES mixed variant (TES-MIXv)

a) Both cognitive core features and behavioral or mood core features (or both).

4) TES dementia (TES-D)

a) Progressive course of cognitive core features with or without behavioral or mood core features (or both).

b) Evidence of ‘functional impairment’, defined as cognitive impairment (or cognitive impairment exacerbated by behavioral or mood impairment or both) that is severe enough to interfere with the ability to function independently at work or in usual activities, including hobbies, and instrumental activities of daily living. The determination of functional impairment is based on clinician’s judgment, taking into account informant reports as well as consideration of individual differences with regard to level of expected responsibility and daily challenges.

c) If the clinical presentation is not distinguishable from that of dementia due to AD or another neurodegenerative disease (for example, frontotemporal dementia), both diagnoses may be given, either with one being ‘primary’ and the other being ‘secondary’ or with the term ‘mixed’ used if neither is presumed primary.

## ‘With motor features’ modifier

For each TES subtype, the modifier ‘with motor features’ should be added if the individual demonstrates dysarthria, dysgraphia, bradykinesia, tremor, rigidity, gait disturbance, falls, and/or other features of parkinsonism.

## Clinical course

For each TES subtype, one of the following additional modifiers should be selected: ‘stable course’, to be used when the history or objective testing (or both) indicates that there has been little if any change in symptoms, signs, or other measures; ‘progressive course’, to be used when there is a clear indication of progressive worsening of clinical features for at least a 2-year period; and ‘unknown/inconsistent course’, to be used when either there is too little information available about the clinical course or the course has been inconsistent, with periods of stability, worsening, and/or improvement. By definition, TES dementia has a progressive course and does not require this modifier.

## ‘Possible CTE’ and ‘probable CTE’

As stated above, CTE is a neuropathological diagnosis, whereas TES is a clinical diagnosis. As with other neurodegenerative diseases, such as AD, it is not possible at this time to diagnose the underlying disease with certainty during life. However, again as with other neurodegenerative diseases and in keeping with the diagnostic criteria for CTE proposed by Jordan [35],[40],[41], we propose provisional diagnostic classifications of ‘probable CTE’, ‘possible CTE’, and ‘unlikely CTE’. Because the scientific study of the clinical presentation of CTE is only in its infancy, it is not yet possible to create meaningful diagnostic criteria for ‘probable CTE’ based solely on clinical features and course, such as those employed for the National Institute on Aging-Alzheimer’s Association (NIA-AA) AD diagnostic criteria for probable AD dementia [60], a condition that has been carefully studied for many decades. Rather, we propose, as a starting point, several potential *in vivo* biomarkers for CTE that can be used to support a provisional diagnosis of ‘probable CTE’. This diagnosis would be analogous to the NIA-AA diagnosis of probable AD dementia with evidence of the AD pathophysiological process [60]. However, because of the early stage of research into potential CTE biomarkers, we refrain from using this type of nomenclature. The following list of potential biomarkers for underlying CTE is meant only as a guideline at this early point in CTE diagnostic research. Many of these biomarkers are the focus of current research but have not yet been formally validated. Future biomarker validation studies will likely add to or delete (or both) items on this list. Moreover, we do not in any way recommend that the specific tests used for these potential biomarkers be conducted for clinical purposes at this time.

## Potential biomarkers for the diagnosis of probable chronic traumatic encephalopathy

1) *Cavum septum pellucidum*. Report of cavum septum pellucidum, cavum vergae, or fenestrations based on neuroimaging study.

2) *Normal beta amyloid cerebrospinal fluid (CSF) levels*. CSF beta amyloid levels in the normal range for age and not diminished as would be suggestive of AD.

3) *Elevated CSF p-tau/tau ratio*. CSF p-tau/total tau ratio above the normal range for age.

4) *Negative amyloid imaging*. PET amyloid imaging (for example, florbetapir and flutemetamol) in the normal range, not suggestive of AD.

5) *Positive tau imaging*. PET paired helical filament tau imaging suggestive of abnormal tau deposition. It should be noted that this remains an experimental procedure and requires additional validation prior to its use as a research tool for diagnostic purposes.

6) *Cortical thinning*. Based on magnetic resonance imaging (MRI) measurement, evidence of abnormal cortical thinning indicative of neurodegeneration.

7) *Cortical atrophy.* Based on MRI or computed tomography, generalized cortical atrophy beyond what is expected for age, and, in particular, frontal, thalamic, hippocampal, and/or amygdalar atrophy.

## Chronic traumatic encephalopathy classification

1) Probable CTE. Meets classification for any TES subtype, progressive course; does not meet diagnostic criteria for another disorder more consistently than TES; and has a minimum of one positive potential biomarker for CTE.

2) Possible CTE. Meets classification for any TES subtype, progressive course, and (1) has not undergone any potential biomarker testing, (2) has had negative results on one or more biomarkers with the exception of PET tau imaging (that is, if a negative PET tau imaging finding, the current classification would be ‘unlikely CTE’), or (3) meets the diagnostic criteria for another disorder that, on its own, could account for the clinical presentation.

3) Unlikely CTE. Does not meet TES diagnostic criteria or has had a negative PET tau imaging scan or both.

Case A A 45-year-old married man with a history of playing multiple contact sports, including soccer (ages 5 to 13), hockey (ages 7 to 12), and football (ages 9 to 22) presented to his primary care physician. He played college football at a Division 1 university and was an offensive lineman. He had no reported or formally diagnosed concussions, although when provided with a definition of concussion, he stated that he likely had 20 to 30 throughout high school and college. Since graduating from college, he has worked as an auditor for state government. His work performance evaluations had been routinely positive, although for the past two years they have been marred by reports of ‘careless errors’, reduced productivity, and one episode of yelling at his immediate supervisor. His wife of 16 years reports that he has had a 5- to 7-year history of worsening behavior, with frequent episodes of having a ‘short fuse’ and losing his temper with their two young children. Though always a social drinker, he has had frequent episodes of binge drinking over the past 2 to 3 years. She states that his personality has changed from a kind, even-keeled, loving man to an argumentative, explosive, and moody individual. Both he and his wife state that he was high-functioning, without any cognitive, mood, and behavioral problems during the time period between college and about age 35. He recently underwent formal neuropsychological evaluation that demonstrated moderately impaired sustained attention, mildly impaired delayed recall on a word list, and moderately impaired executive functioning as measured by a card-sorting test. All other areas of functioning were within the normal range. A self-report measure of syndromal depression indicated mild to moderate severity. Other than the recent work-performance evaluations, there were no other reports of significant functional decline. The result of a recent brain MRI was unremarkable other than some mild, scattered white matter abnormalities. Other medical history, laboratory findings, and neurological examination were unremarkable. Diagnosis: TES-MIXv, progressive course; possible CTE.

Case B A 31-year-old single female Army veteran was referred to the VA Medical Center Behavioral Health Clinic for a 14-month history of suicidal thoughts, agitation, and aggressive behavior. She had reached the rank of staff sergeant and was a logistics specialist. She was honorably discharged 1 year ago, began working in her family’s grocery store, but had to stop working 6 months ago because of her neuropsychiatric symptoms. She had two deployments to Afghanistan and denied being directly involved in combat. However, she reported that 20 months prior to her discharge, she was thrown off a truck when it struck an improvised explosive device. She was told she landed on her head and lost consciousness for 2 to 3 minutes. Upon regaining consciousness, she reported ‘seeing stars’ and had a headache that lasted 3 to 4 days. She denied these symptoms to the medic when questioned and remained on active duty. About 3 months later, a heavy box fell on her head, throwing her to the floor. She denied loss of consciousness but was nauseated and had balance difficulties for several hours. She complained of being in a fog and irritable for 2 days following the accident. Her tour of duty ended 2 weeks later and she returned home. Other than those two injuries, she denied any TBIs or concussions. These symptoms completely cleared, and she described her functioning, including her mood, as ‘completely fine’ between that time and about 14 months ago. Prior to enlisting, she was an avid ice hockey player, having played since the age of 5, and was the captain of her high school team. Her medical and psychiatric histories were unremarkable, and laboratory results of tests ordered by her primary care physician were normal. At the current evaluation, a mental status examination was conducted and the results were generally within normal limits. She denied having any cognitive complaints. A psychiatric interview revealed significant overall distress, with suicidal ideation without any active plan. Her primary complaints included poor sleep, sadness, anxiety, agitation, and being overly aroused by loud noises. She denied having any flashbacks or night terrors. A sibling was interviewed and corroborated the description and history but added that for the past year she had been verbally aggressive and explosive, frequently yelling at family members for no apparent reason, and that these episodes seemed to turn off and on without any warning. The sibling stated that these abnormal behaviors have been somewhat consistent over the past year. A PTSD specialist examined the patient, reported that she would not meet criteria for PTSD, and questioned whether the symptoms were residual from her TBIs in Afghanistan. The result of a brain MRI was unremarkable. Diagnosis: TES-BMv, stable course; possible CTE.

Case C A 59-year-old man presented to his primary care physician with complaints of progressive memory and concentration problems. Prior to going to college, the patient entered the Army, where he boxed competitively for 4 years. He did not experience any combat. He was an avid rugby player in college and continued playing in formal competitive clubs until the age of 54, when he stopped because of a cervical disk injury. He received an MBA and had been a successful business consultant. He was divorced at the age of 45 and lived alone. He reported one concussion at the age of 30, when he briefly lost consciousness during a rugby game, although he stated he got his ‘bell rung’ countless times in boxing and rugby. He reported to his primary care physician that he had been having difficulty remembering details of conversations and meetings at work and that this was beginning to interfere with his productivity. His medical history was significant for the cervical disk injury and for migraine headaches for many years. He was referred to a local academic medical center memory clinic, where a formal neuropsychological evaluation demonstrated moderately impaired performance on a word list recall task, compared with age and education norms, as well as severely impaired fine motor dexterity. All other areas were intact, although his performance on a measure of psychomotor speed and response set maintenance was slightly below expected levels given his history. A neurological examination revealed mild bilateral resting tremor and mild upper extremity rigidity. An MRI scan was read as normal, and all laboratory findings were within normal limits. As part of a clinical research study, he was given two PETs: one with a new tau radiotracer and another with an amyloid tracer. Results indicated no meaningful amyloid uptake, although his tau scan was abnormal with scattered increased tracer uptake in the dorsolateral frontal cortex and the medial temporal lobes. Diagnosis: TES-COGv, with motor features, progressive course; probable CTE.

Case D A 69-year-old former National Football League (NFL) football player was seen in consultation following a 10-year progressive decline. He had seen several physicians and had been given multiple diagnoses, including frontotemporal dementia and dementia due to AD. He had played professional football for 9 years as a linebacker. He began playing football in high school and played for a Division 1 college for 4 years, playing both as a linebacker and as an offensive lineman. Following retirement from the NFL, he had a successful career in commercial real estate until he was forced to retire at the age of 62 because of ‘poor decision-making and judgment’. His wife of 25 years stated that, in retrospect, he was demonstrating poor memory and judgment for about 3 years prior to his retirement and that these problems had progressively worsened through the years. She stated that he also began having significant difficulties with multi-tasking and ‘numbers’ at age 61 and was having difficulty with household finances and hobbies. After retirement, he became increasingly withdrawn and refused to socialize. In contrast to his previous jovial and easy-going manner, he became verbally aggressive toward his wife and children, ‘blowing up over small things’. On two occasions, he became physically aggressive toward his wife, requiring her to call the police. He never demonstrated any disinhibited or socially inappropriate behavior, nor was there any report of hallucinations or movement disturbance. In the past 2 years, his functioning has worsened; he now has no ‘short-term memory’, watches television all day long, and has an erratic sleep cycle. He is functionally impaired in all instrumental activities of daily living as well as in some basic activities of daily living. His medical history is significant for a myocardial infarction at age 54, hypertension, severe arthritis, and multiple lumbar disk surgeries. There is no family history of dementia. Upon examination, he was disoriented to time and place, was perseverative, and could not recall recent current events. He exhibited some frontal release signs, although the result of his motor examination was otherwise normal. His Mini-Mental Status Exam score was 9, and his Clinical Dementia Rating was 2.0. A neuropsychological evaluation was conducted and demonstrated severe episodic memory impairment as well as profoundly impaired performance on most tests of executive functioning. In contrast, attentional capacity was within normal limits and language was relatively intact. A brain MRI revealed significant global atrophy with marked hippocampal atrophy as well as a cavum septum pellucidum. An amyloid PET scan demonstrated only minimal uptake, not commensurate with the degree of dementia. Diagnosis: TES-D; probable CTE.

Case E A 31-year-old male stockbroker saw his primary care physician because of an 18-month history of recurrent headaches, irritability, agitation, and a worsening ‘short fuse’. He had been taking oxycodone (left over from previous oral surgery) for his headache pain. He was referred to a neurologist, who specialized in headache and who diagnosed him with tension headache. However, when asked if he had ever had headaches previously, the patient reported that he frequently had them as a teenager after his varsity high school football games and when he played rugby for 2 years in college. Because of this history of prior exposure to repetitive head impacts and possible symptomatic concussions, the neurologist referred him to a psychiatrist colleague to evaluate him for possible depression and suicidality, based on the neurologist’s belief that the patient might have CTE; he had recently attended a talk on sports injuries. The consulting psychiatrist interviewed the patient, who acknowledged that he had frequent suicidal ideation following the breakup of his marriage about 1 year earlier but that these thoughts had now diminished. Although the patient formally met criteria for TES-BMv, the psychiatrist felt that the headache symptoms, suicidality, short fuse, and irritability were likely associated with the divorce. The patient was prescribed citalopram as well as regular therapeutic massage for his tension headache and was seen in 3 months, at which time he reported substantial improvement of his mood and behavioral symptoms and a complete resolution of his headaches. Diagnosis: adjustment disorder, persistent with mixed anxiety and depressed mood; unlikely CTE.

Case F An 81-year-old widowed man enrolled in a research study examining the long-term consequences of TBI. He reported having sustained a moderate TBI in a motor vehicle accident at the age of 46 with loss of consciousness for approximately 1 hour. He was hospitalized for 3 days because of confusion and memory difficulties that mostly resolved prior to discharge. He was unable to return to work as a high school physical education teacher and coach for several weeks because of continued cognitive difficulties, headache, and balance problems. He reported that, once he returned to work, he ‘didn’t feel normal’ for several months. He continued working until retirement at age 60. He played high school and college football and reported having had his ‘bell rung’ ‘all the time’. According to his adult son (with whom he lived), he was 72 when he began having memory problems that gradually progressed over the course of 5 to 6 years. In the past few years, the memory problems worsened significantly, such that he could not recall events that occurred more than an hour earlier. In addition, he had worsening problems with judgment, decision-making, multi-tasking, and word-finding. He no longer drove and was dependent in most areas of instrumental activities of daily living. He lacked interest in all activities and appears ‘depressed’ according to his son. His medical history was significant for prostate cancer, controlled hypertension, arthritis, and glaucoma. Two brothers died in their 80s with ‘dementia’. Neuropsychological testing revealed significant impairments in episodic memory, confrontation naming, psychomotor speed, and many aspects of executive functioning. Research-based MRI revealed frontal and temporal atrophy and a pronounced cavum septum pellucidum; diffusion tensor imaging and tractography demonstrated significant reductions in corpus callosum fiber bundles. PET amyloid imaging showed elevated uptake consistent with AD. Diagnosis: dementia due to AD pathophysiological process and TES-D, mixed; possible CTE.

The current proposed research diagnostic criteria for TES are meant to be a starting point that should be modified and updated as new research findings in the field become available and as future research using these criteria are published. These proposed criteria are not meant to be used for a clinical diagnosis or as evidence of an underlying disease. Rather, they should be viewed as research criteria that could be employed in studies of the underlying causes, risk factors, differential diagnosis, prevention, and treatment of TES. Future studies comparing these proposed diagnostic categories with post-mortem neuropathological diagnoses, as well as with appropriate *in vivo* biomarkers for CTE and other conditions, will help lead to the transition from ‘research’ criteria to ‘clinical’ criteria. It also would be critical for these proposed criteria to undergo a formal expert consensus approval process, such as that used for the NIA-AA Diagnostic Guidelines for Alzheimer’s Disease [60].

One important factor that must be addressed in future iterations of these criteria is that of base rates. That is, the population prevalence of most of the core clinical features and many of the supplemental features of TES presented below is relatively high. Therefore, it is possible to meet criteria for TES and yet have an idiopathic disorder or a situationally based condition that is unrelated to the earlier history of head impact exposure. The inclusion of supportive features is meant to reduce this lack of specificity to a degree, but, at this time, we acknowledge that these criteria will likely result in very high sensitivity at the expense of specificity. With the utilization of future research findings and subsequent criteria revisions, it is likely that the specificity will increase. An important additional issue regarding the use of these criteria involves the impact of litigation or disability determination (or both) on the validity of symptom reporting and neuropsychological test performance. It is therefore recommended that this issue be taken into account when interpreting the individual’s self-reported functioning and test performance and that formal symptom validity checking be conducted as part of any formal evaluation. Until future research yields accurate biomarkers and allows clarification and modification of the proposed criteria, the decision as to whether an individual meets the TES diagnostic criteria and associated ‘probable CTE’ diagnostic criteria should be left up to the individual researcher, clinician, or, preferably, a multidisciplinary diagnostic adjudication process.

## Conclusions

The long-term consequences of repetitive head impacts have been known since the beginning of the 20th century. Although the clinical presentation of CTE is varied and non-specific, there are adequate reports to date to suggest that there may be two clinical subtypes: one subtype involving primarily behavioral or mood features (including explosivity or violence) or both, and the other involving cognitive deficits (including impairments in episodic memory, executive functioning, and attention). Many individuals progress to dementia, with impaired functional independence, and some individuals develop motor impairments (including parkinsonism, ataxia, and dysarthria). We propose research diagnostic criteria for TES that we hope will facilitate research into this area. There are expected limitations to the development of diagnostic criteria based primarily on a relatively small number of case reports. The goal of proposing these criteria at this time is to facilitate research in this nascent area of study. It is expected that these criteria will undergo modification and revision as new research findings become available, additional biomarkers are validated, and future research using these criteria are published.

**Note**

This article is part of a series on *Traumatic brain injury*, edited by Robert Stern. Other articles in this series can be found at http://alzres.com/series/traumaticbraininjury

## Abbreviations

AD: Alzheimer’s disease

CSF: Cerebrospinal fluid

CTE: Chronic traumatic encephalopathy

MRI: Magnetic resonance imaging

NFL: National Football League

NIA-AA: National Institute on Aging-Alzheimer’s Association

PET: Positron emission tomography

pPCS: Persistent post-concussion syndrome

p-tau: Phosphorylated tau

PTSD: Post-traumatic stress disorder

TBI: Traumatic brain injury

TE: Traumatic encephalopathy

TES: Traumatic encephalopathy syndrome

TES-BMv: Traumatic encephalopathy syndrome behavioral/mood variant

TES-COGv: Traumatic encephalopathy syndrome cognitive variant

TES-D: traumatic encephalopathy syndrome dementia

TES-MIXv: Traumatic encephalopathy syndrome mixed variant

## Competing interests

AEB receives royalties for published books from Elsevier (Amsterdam, The Netherlands) and Wiley-Blackwell (Hoboken, NJ, USA). RCC receives compensation from the NFL as senior advisor to the Head Neck and Spine Committee, from the National Operating Committee on Safety of Athletic Equipment as chairman of the Scientific Advisory Committee, and from Sports Legacy Institute as co-founder and medical director for some talks given. He receives royalties from Houghton Mifflin Harcourt (Boston, MA, USA) and compensation from expert legal opinion. RAS has received research funding from the NFL and the NFL Players Association. He is a member of the Mackey-White Traumatic Brain Injury Committee of the NFL Players Association. He is a paid consultant to Athena Diagnostics (Marlborough, MA, USA) and has been a consultant to Janssen Alzheimer Immunotherapy (South San Francisco, CA, USA) and Ely Lilly and Company (Indianapolis, IN, USA). He receives royalties for published neuropsychological tests from Psychological Assessment Resources, Inc. (Lutz, FL, USA) as well as compensation from expert legal opinion. The other authors declare that they have no competing interests.
